# Disruption of Dense Granular Protein 2 (GRA2) Decreases the Virulence of *Neospora caninum*

**DOI:** 10.3389/fvets.2021.634612

**Published:** 2021-02-19

**Authors:** Jingquan Dong, Nan Zhang, Panpan Zhao, Jianhua Li, Lili Cao, Xiaocen Wang, Xin Li, Ju Yang, Xichen Zhang, Pengtao Gong

**Affiliations:** ^1^Key Laboratory of Zoonosis, College of Veterinary Medicine, Jilin University, Changchun, China; ^2^Jiangsu Key Laboratory of Marine Biological Resources and Environment, Jiangsu Key Laboratory of Marine Pharmaceutical Compound Screening, Co-Innovation Center of Jiangsu Marine Bio-industry Technology, Jiangsu Ocean University, Lianyungang, China; ^3^Department of Parasite, Jilin Academy of Animal Husbandry and Veterinary Medicine, Changchun, China

**Keywords:** *Neospora caninum*, CRISPR, dense granules protein 2, intravacuolar network, immunofluorescence

## Abstract

*Neospora caninum* causes abortions in cattle and nervous system dysfunction in dogs. Dense granular proteins (GRAs) play important roles in virulence; however, studies on NcGRA functions are limited. In the present study, multiple methods, including site-directed mutagenesis; CRISPR/Cas9 gene editing; Western blotting; quantitative polymerase chain reaction; confocal microscopy; plaque, invasion, egress, and replication assays; animal assays of survival rate and parasite burden; and hematoxylin–eosin staining, were used to characterize the NcGRA2 protein, construct an NcGRA2 gene disruption (ΔNcGRA2) strain, and explore its virulence *in vivo* and *vitro*. The results showed that NcGRA2 shared 31.31% homology with TgGRA2 and was colocalized with NcGRA6 at the posterior end of tachyzoites and the intravacuolar network of parasitophorous vacuoles (PVs). Cell fractionation analysis showed that NcGRA2 behaved as a transmembrane and membrane-coupled protein. The ΔNcGRA2 strain was constructed by coelectroporation of the NcGRA2-targeting CRISPR plasmid (pNc-SAG1-Cas9:U6-SgGRA2) and DHFR-TS DNA donor and verified at the protein, genome, and transcriptional levels and by immunofluorescence localization analysis. The *in vitro* virulence results showed that the ΔNcGRA2 strain displayed smaller plaques, similar invasion and egress abilities, and slower intracellular growth. The *in vivo* virulence results showed a prolonged survival time, lower parasite burden, and mild histopathological changes. Overall, the present study indicates that NcGRA2, as a dense granular protein, forms the intravacuolar network structure of PVs and weakens *N. caninum* virulence by slowing proliferation. These data highlight the roles of NcGRA2 and provide a foundation for research on other protein functions in *N. caninum*.

## Introduction

*Neospora caninum*, an obligate intracellular protozoan pathogen, belongs to the phylum Apicomplexa and leads to neosporosis with clinical manifestations of reproductive failure in bovines and nervous system dysfunction in dogs (1–7). It has been reported that the total annual loss in the cattle industry caused by *N. caninum* infection worldwide is as high as US $43.08 billion to US $320.98 billion (8, 9). As a country with a large amount of animal husbandry, China has begun to pay increasing attention to *N. caninum* infections. Currently, no commercial treatment with *N. caninum* is available. Pretreatment and control of neosporosis are mainly by sulfonamides or attenuated vaccines; however, these drugs have relatively large negative effects, and resistance to them forms easily, leading to incomplete elimination of *N. caninum* (10, 11). Thus, it is urgent to look for new targets against neosporosis.

*N. caninum* replicates in a specialized membranous organelle known as the parasitophorous vacuole (PV), which is formed by parasites inside host cells after invasion, separates parasites from the cytoplasm, and resists host cell acidification and lysosomal zymohydrolysis (12). The intravacuolar network (IVN) inside the PV is composed of highly curved membrane tubules and shares the functions of ingesting nutrients from hosts and linking parasites and the PV membrane (PVM). Similar to other apicomplexan parasites, *N. caninum* discharges proteins produced in secretory organelles, and these are micronemes (MICs), rhoptries (ROPs) and dense granules (GRAs) during periods of invasion into host cells, adhesion to the surface of cell membranes, or survival and development in the PV (13). MICs are not only involved in parasite attachment to hosts, but also play important roles in invasion. ROPs are restricted to the bulb of organelles and secreted while/after the formation of the PVs to manipulate the host cell. RONs are from the neck of ROPs and play roles in parasite attachment and invasion into hosts (14). Most of the information about the roles of MICs, ROPs, and GRAs comes from *Toxoplasma gondii*, which is closely related to *N. caninum*. GRAs are involved in remodeling and maintaining the environment of the PV and play key roles in the replication of *T. gondii* (15–18).

The IVN is the most important structure within the PV in *T. gondii*. Apart from the aforementioned nutrient absorption and establishment connection with PVM, the IVN can also help *T. gondii* to divide in order to optimize their synchronous division (19–21) and to deliver secreted proteins to construct the PVM (22, 23) and is involved in virulence by accelerating ROP release into PVM and then maintain PVM stabilization (22, 24). Furthermore, TgGRA2 is regarded as a typical GRA candidate involved in IVN biogenesis. It is embedded in the IVN through the structures of two amphipathic alpha helices (AAHs) (21). Deletion of the TgGRA2 gene completely disrupts the IVN structure, and complementation assays of TgGRA2 proteins further showed that not only the AAH structure but also the N-terminal hydrophilic domain were important factors in the formation of IVN (21, 25). Despite these clues verifying the importance of TgGRA2, GRA2 in *N. caninum* was first identified in 2000; its location in bradyzoites was explored in 2004, and its immune protection was evaluated in a mouse model using recombinant GRA2 protein in 2008; however, its role in *N. caninum* virulence is still unknown (26–28).

In *N. caninum*, studies on NcGRA functions are limited. Here, we characterized an *N. caninum* GRA protein, NcGRA2. Using the *N. caninum–*specific CRISPR/Cas9 system, we generated an NcGRA2 disruption strain (ΔNcGRA2) based on the Nc-1 strain of *N. caninum*; verified the ΔNcGRA2 strain at the protein, genome, and transcriptional levels; and, through localization analysis, and further explored its pathogenicity *in vivo* and *in vitro*. These data highlight the roles of NcGRA2 in *N. caninum* virulence.

## Materials and Methods

### Ethics Statement

All animal experiments have received approval for research ethics from the Animal Welfare and Research Ethics Committee of Jilin University, and the certificate number is pzpx20190929065. Five-week-old BALB/C female mice and 6-month-old NZW male rabbits were fed sterile water and food and housed in 12-h light–dark cycle feeding cages.

### Cells and Parasite Strains

Bovine kidney epithelial (MDBK) cells and human skin fibroblast (HFF) cells (ATCC, USA) were cultured in Dulbecco modified eagle medium (DMEM; Biological Industries, Israel) supplemented with 10% fetal bovine serum (FBS; Biological Industries, Israel), 100 U/mL penicillin, and 100 μg/mL streptomycin (Biological Industries, Israel) under conditions of 37°C and 5% CO_2_. MDBK cell line shares fast growth rate; in contrast, HFF cell line shares relative slow growth rate. MDBK cells can be passaged every 2 days in a ratio of 1:3 and HFF cells can be passaged every 5–7 days in a ratio of 1:2. For *N. caninum* tachyzoites preparation, it is better to use MDBK cell line to obtain enough tachyzoites with a shorter period. For plaque assays used in the construction of the ΔNcGRA2 strain and virulence *in vitro*, HFF cell line is a better choice for its slow growth rate, makes single tachyzoite form observed plaques, and increases the success probability of single tachyzoite plaque screening. Meanwhile, considering the necessary of HFF cell line for plaque assays, other virulence studies *in vitro* were also used in the HFF cell line.

To rapidly obtain the Nc-1 strain of *N. caninum* tachyzoites, tachyzoites were grown in fast-growing MDBK cells and purified as described previously (29). In brief, *N. caninum* tachyzoites were collected when 80% of the host cells were lysed and passed through a 27-gauge needle. Then, the tachyzoites were isolated from host cell debris and further purified with 40% (vol/vol) Percoll (GE Healthcare, Uppsala, Sweden) by centrifugation at 2,500 × *g* for 10 min. After washing with phosphate-buffered saline (PBS), the concentrations of tachyzoites were determined with a hemocytometer.

### Preparation of a Rabbit Polyclonal Antibody Against NcGRA2

The NcGRA2 gene was amplified from tachyzoite cDNA with specific primers (forward: 5′-ATG*GAATTC*GCCGATTTTTCTGGCAGGGGAA-3′; reverse: 5′-GTA*CTCGAG*TTAATTGACTTCAGCTTCT-3′) using Phusion® High-Fidelity DNA Polymerase (NEB, Inc., USA) according to the manufacturer's instructions. The underlined nucleotides in the forward and reverse primer are the restriction enzyme cutting sites of *EcoR*I and *Xho*I, respectively. The purified NcGRA2 polymerase chain reaction (PCR) products and extracted pGEX-4T-1 plasmid were treated with *EcoR*I and *Xho*I enzymes and ligated together with T4 DNA ligase (TaKaRa, Dalian). The mixtures were transformed into Trans1T1 competent cells (TranGen, Beijing), and the recombinant plasmid pGEX-4T-NcGRA2 was identified through sequencing. The positive pGEX-4T-NcGRA2 plasmid was transformed into BL21 competent cells (TranGen, Beijing), followed by induction of expression. The recombinant pGEX-4T-NcGRA2 protein (rNcGRA2) was analyzed using sodium dodecyl sulfate–polyacrylamide gel electrophoresis (SDS-PAGE). The soluble rNcGRA2 protein was purified using GSTrap FF purification columns (GE Healthcare Bio-Sciences, USA). NZW rabbits were immunized subcutaneously with 500 ng of rNcGRA2 emulsified with equal volume of Freund's complete adjuvant (Sigma, USA). The rabbits were boosted with 300 ng of rNcGRA2 emulsified with equal volume of Freund's incomplete adjuvant two times with a 14-day interval. Polyclonal antibodies against rNcGRA2 were generated after 14 days since the last injection.

### Plasmid Construction of the NcGRA2 CRISPR Gene Editing Vectors

An *N. caninum* gene editing plasmid, pNc-SAG1::CAS9-U6::sgUPRT (Supplementary Figure 1), which contained the *N. caninum* U6 promoter, Cas9-monomeric enhanced green fluorescent protein (Cas9-mEGFP), 5′ UTR and 3′ UTR of the *N. caninum* SAG1 gene, sgRNA sequence of UPRT, ampicillin resistance gene (AmpR), origin of replication (ori), and f1 ori, was used as a template to construct the NcGRA2 CRISPR gene editing vectors (29). Specific sgRNA sequences were searched via E-CRISP online software (http://www.e-crisp.org/E-CRISP/designcrispr.html), and primer sequences for sgNcGRA2 were designed, which are listed in Table 1. The primer sgNcGRA2-F contained an extra 20 bp of guide RNA sequence for NcGRA2 and 20 bp of nucleotide sequence adjacent to the guide RNA in pNc-SAG1::CAS9-U6::sgUPRT. To substitute sgUPRT with sgNcGRA2, site-directed mutagenesis was carried out using a Q5® Site-Directed Mutagenesis Kit (NEB, USA) according to the manufacturer's instructions. The amplification products were verified using sequencing methods by Comate Bioscience Company.

**Table 1:** Primer sequences used for generation of the ΔNcGRA2 strain.

**Primer name**	**Sequence (5^′^-3^′^)**
sgNcGRA2-F^a^	GGTTATTCCGGATATCCCCGGTTTTAGAGCTAGAAATAGC
sgNcGRA2-R^b^	AAACAACAATGTCCCTTTGGCA
KO^c^-NcGRA2-F	ATGTTCACGGGGAAACGTTGGATATAAGCTTTACTCGTCGCCAGCAGT
KO^c^-NcGRA2-R	CTATTTTTCCTCCCCGCCGTTTTCGGTCGGAATTTAGGTCGGAAAAGT

a *F represented forward*.

b *R represented reverse*.

c *KO represented knockout*.

### Construction of the ΔNcGRA2 Strain

The DHFR-TS DNA donor was amplified using the pNcDHFR plasmid as a template. After purification, 2 μg of DHFR-TS DNA donor, 6 μg of pNc-SAG1::CAS9-U6::sgGRA2 plasmid, and 300 μL of Nc-1 strain [4 × 10^7^ tachyzoites/mL dissolved in Cytomix (Thermo Fisher, USA)] were fully mixed and then electroporated under conditions of 1,500 V/25 μF/50 Ω. Next, the transfection mixture was inoculated into MDBK cells and cultured under conditions of 37°C and 5% CO_2_. Tachyzoites were collected when 80% of the host cells were lysed, and the *N. caninum* ΔNcGRA2 strain was screened as follows. First, 2 mL of purified tachyzoites was inoculated into slow-growing HFF cells and cultivated for 7 h. Then, 1 μM pyrimethamine was added into the cultures, which were incubated for 5–7 days until half of the tachyzoites had egressed (30). After purification, tachyzoites were again inoculated into HFF cells and screened under pyrimethamine. Next, a total of 80–120 tachyzoites were suspended in 10 mL of DMEM supplemented with 10% FBS and inoculated into a 96-well plate at 100 μL per well to make tachyzoites replication (none, one or more tachyzoites per well). After culturing for 5 days, the plate was observed under an optical microscope; wells with no or multiple plaques were discarded, and wells with a single plaque were continuously cultured for another 3 days. Then, the single tachyzoites in wells with a single plaque were transferred to HFF cells cultured in 12-well plates. The screened ΔNcGRA2 strain was then assessed using Western blotting detection, PCR amplification, real-time quantitative PCR (qPCR) analysis, and immunofluorescence observation.

### Protein Sample Preparation, SDS-PAGE, and Western Blotting

Analysis of the major forms of the NcGRA2 protein and assessment of the ΔNcGRA2 strain at the protein expression level were both carried out using Western blotting assays. For NcGRA2 protein form analysis, infected MDBK cells were resuspended in PBS, and supernatants were collected after elimination of parasites and cell debris by needles and centrifugation at 2,500 × *g* for 10 min. Next, cell fractionation was used to divide the samples into soluble [high-speed supernatant (HSS)] and membrane-associated [high-speed pellet (HSP)] fractions by ultracentrifugation at 100,000 × *g* for 2 h. HSPs were dissolved in 50 μL of 50 mM Tris buffer (pH 8.0) supplemented with 1 mM PMSF (Solarbio, China) protease inhibitor. The HSS was precipitated with acetone (31), and the pellets were suspended in an equal volume of Tris buffer used in the HSP sample. To further separate proteins, the HSP solution was sonicated (60 H/30 s) on ice for 30 min and stored in a denaturant of 10% Triton X-114. Finally, the samples were collected and ultracentrifuged at 100,000 × *g* for 2 h to separate the pellets and supernatants. The obtained detergent phase (D) and aqueous phase (A) were individually precipitated with acetone and dissolved in an equal volume of Tris buffer (32). The prepared samples (10 μL) were used for SDS-PAGE. For assessment of the ΔNcGRA2 strain, total proteins were extracted from the Nc-1 and ΔNcGRA2 strains using RIPA lysis buffer containing 1 mM PMSF protease inhibitor. Concentrations of these obtained proteins were measured using a BCA Protein Assay Kit (Thermo Scientific, USA), and 20 μg of protein samples was used for SDS-PAGE. Protein samples were mixed with 5 × SDS-PAGE sample loading buffer (Beyotime, China), boiled for 10 min, subjected into 12% SDS-PAGE, and then electrophoresed in Tris–glycine–SDS buffer under conditions of 80 V for 1 h and then 120 V for 40 min. For Western blotting analysis, the targeted proteins were transferred onto 0.45 μm PVDF membranes (Millipore, USA) under conditions of 200 mA for 1 h. The membranes were blocked in 5% skim milk at 4°C overnight and then incubated with a specific rabbit polyclonal antibody against NcGRA2, a specific rabbit polyclonal antibody against NcGRA7, or a mouse polyclonal antibody against NcSRS22A (prepared in our laboratory) at a dilution of 1:200 under conditions of 37°C for 1 h. NcGRA7 antibody was used to verify the purity of fractions. NcSRS22A is a putative surface antigen and used as a control in *N. caninum*. After washing three times (5 min per wash) with PBST, the membranes were then incubated with secondary antibodies of either goat anti-rabbit immunoglobulin G (IgG) (H+L) or goat anti-mouse IgG (H+L) (Earthox, USA) at a dilution of 1:5,000 under conditions of 37°C for 1 h. Finally, the membranes were washed three times again with PBST and incubated with ECL chemiluminescence reagents (Thermo Scientific, USA) for 5 min. The bands were viewed on a Western Blot Imaging System (Clinx, China).

### PCR Amplification

Amplification of the DHFR-TS DNA donor or assessment of ΔNcGRA2 was conducted through PCR assays with Phusion® High-Fidelity DNA Polymerase according to the manufacturer's instructions. For DHFR-TS DNA donor amplification, specific primers for KO-NcGRA2 were designed and are listed in Table 1. The primer set for KO-NcGRA2 contained 23 bp of homologous sequences with the two ends of the protospacer adjacent motif in the *N. caninum* genome at the 5′ end and 25 bp of specific DHFR-TS sequences at the 3′ end. The aim of introduction with homologous sequences was to make homology-directed repair (HDR) and non-homologous end joining (NHEJ) simultaneously function and finally improve the recombinant efficiency. Errors in annealing may probably be due to incompletely matched at the 3′ end, and unmatched sequences were introduced at the 5′ end in the present study. For ΔNcGRA2 assessment, specific primers targeting the 5′ UTR and 3′ UTR of the *N. caninum* SAG1 gene (P1 and P2) or DHFR-TS gene (P3 and P4) were designed and are listed in Table 2. The reaction system contained 200 ng plasmid template of pNcDHFR or 1 μg genome DNA template of ΔNcGRA2 or Nc-1 strains, 0.2 μL of Phusion DNA Polymerase, 4 μL of 5 × Phusion HF Buffer, 0.4 μL of dNTPs (10 μM), 1 μL of forward and reverse primers (10 μM), and nuclease-free water to a final volume of 20 μL. The amplification program was set as follows: predenaturation at 98°C for 30 s, followed by 35 amplification cycles of denaturation at 98°C for 10 s, annealing at 55°C for 30 s, and extension at 72°C for 1 kb/min, with a final extension at 72°C for 10 min. The amplification products were detected on a 1.5% agarose gel.

**Table 2:** Primer sequences used for verification of the ΔNcGRA2 strain.

**Primer name**	**Sequence (5^**′**^-3^**′**^)**
P1	ACAAAGCCCAGGACATCCGAAAA
P2	TCCCACAAGTAACTGTTTTGACTATTT
P3	GAAGCACACGTTTCAGAGACCA
P4	TAGCTCCAGTGTGTCTGTTCCT
NcGRA2-F^a^	CAATGGACAGCCGGTTGGCA
NcGRA2-R^b^	TCCGACCTTCGCAGTAAACT
Actin-F	TGAGAGAGGATACGGTTT
Actin-R	GGCAGCGGAAGCGCTCGTT

a *F represents forward*.

b *R represents reverse*.

### Real-Time qPCR

To assess the ΔNcGRA2 strain, qPCR assays were carried out to measure the GRA2 gene transcription level as previously described (28). Briefly, RNA was extracted from ΔNcGRA2 or Nc-1 strains using TRIzol reagent (Invitrogen, USA) according to the manufacturer's instructions. The parameters of concentration and purity were evaluated on a Nanodrop ND-2000 apparatus (Thermo Scientific, USA). Then, 1 μg of total RNA was used for cDNA synthesis with the PrimeScript™ RT Reagent Kit (TaKaRa, China). qPCR assays were used for quantification of the GRA2 mRNA expression levels with FastStart Universal SYBR Green Master Mix (Roche, Germany) on a qTOWER 2.0 (Analytikjena, Germany) instrument. A total volume mixture of 20 μL contained 10 μL of 2 × SYBR qPCR mix, 1 μL of NcGRA2 or actin primer (10 μM, Table 2), 1 μL of diluted cDNA samples, and nuclease-free water. qPCR was performed as follows: predenaturation at 95°C for 10 min, followed by 40 amplification cycles of denaturation at 95°C for 10 s and annealing and extension at 60°C for 30 s. The fluorescence signal was read at the extension stage after each cycle, and the melting curve was set as the default. The mRNA fold change was measured by calculating 2^−ΔΔCt^, where ΔCt represents the Ct (target gene) – Ct (actin), and ΔΔCt represents the ΔCt (sample) – ΔCt (control).

### Confocal Microscopy

Localization of NcGRA2 protein in the Nc-1 strain of *N. caninum* tachyzoites or infected MDBK cells and assessment of the protein expression levels in the ΔNcGRA2 strain were both performed through immunofluorescence assays. Purified Nc-1 tachyzoites were added to coverslips pretreated with 0.1 mg/mL poly-l-lysine (Sigma, St. Louis, MO, USA) and allowed to stand for 30 min. To clearly locate the distribution of NcGRA2 protein in PVs, the cells previously coated on coverslips were infected with tachyzoites (parasite-host ratio, MOI = 3) and incubated for 48 h. The tachyzoites or infected cells were then fixed in a 4% paraformaldehyde solution at room temperature for 20 min and permeabilized in 0.5% Triton-X-100. After washing three times with PBST, the samples were blocked in 5% bovine serum albumin (BSA) at 4°C for 12 h. For the localization analysis of the NcGRA2 protein, tachyzoites or infected cells were coincubated with a specific rabbit polyclonal antibody against NcGRA2 and a rat polyclonal antibody against NcGRA6 at a dilution of 1:200 under conditions of 37°C for 2 h. Then, cells or tachyzoites were washed three times with PBST and again coincubated with labeled secondary detection antibodies of fluorescein isothiocyanate (FITC)–conjugated goat anti-rabbit IgG (H+L) and Cy3-conjugated goat anti-rat IgG (H+L) (Earthox, USA) at a dilution of 1:400 under conditions of 37°C for 1 h. To assess whether NcGRA2 was successfully disrupted in the ΔNcGRA2 strain, MDBK cells were infected either with the Nc-1 strain or ΔNcGRA2 strain and incubated for shorter time of 24 h. After fixation, the cells were both coincubated with a specific rabbit polyclonal antibody against NcGRA2 and a mouse polyclonal antibody against NcSRS22A at a dilution of 1:200 under conditions of 37°C for 2 h. Subsequently, the samples were again coincubated with labeled secondary detection antibodies of FITC-conjugated goat anti-mouse IgG (H+L) and Cy3-conjugated goat anti-rabbit IgG (H+L) (Earthox, USA) at a dilution of 1:400 under conditions of 37°C for 1 h. Nuclei in the cells and tachyzoites were stained with DAPI (Thermo, USA) at room temperature for 10 min at a final concentration of 300 nM. After washing five times with PBST, tachyzoites or infected cells were analyzed on an FV1000 laser scanning confocal microscopy (LSCM, Olympus, Japan).

### Plaque Assay

Plaque assays were performed as previously described (33). Parasites were seeded onto HFF cells previously coated on six-well plates to a final concentration of 80–120 tachyzoites per well and incubated under conditions of 37°C and 5% CO_2_ for 7 days. Then, the culturing medium was discarded, and the cells were washed three times with PBS and fixed in 4% paraformaldehyde at room temperature for 20 min. After washing three times with PBS, the cells were further stained for 15 min with 1% crystal violet, which was removed with deionized water. The dried plaques were scanned with a light microscope (Olympus, Japan), and the area was calculated as described previously (34).

### Invasion Assay

Invasion assays were performed in HFF cells coated on coverslips in six-well plates. Freshly purified parasites were seeded onto HFF monolayers to a final concentration of 1 × 10^5^ tachyzoites per well and incubated for 20, 40, and 60 min. After fixation, the cells were blocked with 5% BSA at 37°C for 1 h and then incubated with a primary antibody against NcSRS22A at 37°C for 2 h. After washing three times with PBST, the cells were further incubated with a secondary antibody of FITC-conjugated goat anti-mouse IgG (H+L). Next, the cells were permeabilized with 0.2% Triton X-100 at 37°C for 0.5 h and again blocked in BSA at 37°C for 1 h. The cells were incubated sequentially with a specific antibody against NcSRS22A and a secondary detection antibody of Cy3-conjugated goat anti-mouse IgG (H+L), and labeled parasites were observed on the LSCM. The invasion percentage was calculated as {(number of Cy3-labeled parasites – number of FITC-labeled parasites)/number of Cy3-labeled parasites} × 100%.

### Egress Assay

Similar to *T. gondii, N. caninum* tachyzoites replicate in the PV after invasion into host cells and then egress out and enter uninfected cells (35, 36). The egress assays were performed as previously described (29). A total of 1 × 10^5^ tachyzoites were inoculated onto HFF cells coated on coverslips in six-well plates under conditions of 37°C and 5% CO_2_ for 1 h, and then the extracellular parasites were discarded by washing three times with sterilized PBS. HFF cells were cultured in fresh medium for 48 h. Next, egress was stimulated by adding the Ca^2+^ ionophore A23187 (Sigma, USA) to a final concentration of 3 μM for 3 min, and the dimethyl sulfoxide (DMSO)–treated group was used as a control. Immunofluorescence assays were performed using mouse polyclonal antibodies against NcSRS22A and CY3-conjugated goat anti-mouse IgG (H+L) as described above. The disrupted or integrated PV was determined by the distribution of labeled tachyzoites where gathered tachyzoites were in the integrated PV, whereas diffused tachyzoites were found around the disrupted PV. The egress percentage was determined by counting the mean disrupted PV number from 100 randomly selected vacuoles.

### Replication Assay

Purified parasites (1 × 10^6^ tachyzoites per well) were inoculated onto HFF cells coated on coverslips in six-well plates for 4 h, and the supernatants were replaced with fresh medium. Then, cells were collected at different time points of 6, 12, 24, and 36 h; fixed in 4% paraformaldehyde; and used for immunofluorescence assays with antibodies against NcSRS22A and FITC-conjugated goat anti-mouse IgG (H+L) as outlined above. The replication ability was determined by observing the numbers of labeled parasites in 100 randomly selected vacuoles.

### Virulence in Laboratory Mice

Acute infection models and non-lethal infections were both established to evaluate virulence *in vivo*. Five-week-old female BALB/C mice were divided into three groups to explore the difference in virulence between the Nc-1 strain and ΔNcGRA2 strain. For the acute infection models, 2 × 10^7^ tachyzoites were injected intraperitoneally (i.p.) into mice (five mice per group) and monitored for 40 days. Sterilized PBS was used as a control. Mice that lost their ability to drink water or eat food for more than 24 h and lost 20% body weight were humanely euthanized by cervical dislocation after subcutaneous injection of atropine (0.02 mg/kg), and the survival time was recorded (33).

To explore the effects of *N. caninum* on host tissues, a non-lethal dose of tachyzoites (2 × 10^6^ per mouse) was injected i.p. into mice (nine mice per group), and heart, liver, spleen, lung, kidney, and brain tissues were isolated after euthanasia at 3, 7, and 15 days postinfection (dpi). Tissues in the NC group and Nc-1 group were isolated at 15 dpi. The parasite burden was determined by measuring the Nc5 gene contents using an absolute qPCR assay with tissue genomic DNA as a template (37). The histopathological changes in the isolated tissues were determined by hematoxylin–eosin (HE) assay as previously described (29). Briefly, fresh tissues were washed with 0.9% NaCl, cut into patches, and immediately fixed in 4% paraformaldehyde. After dehydration with ethanol and dimethyl benzene, the tissues were embedded in paraffin, sectioned into 5-μm-thick sections, and then mounted on slides. After removal of paraffin and sequential treatment with hematoxylin solution, acid alcohol, and ammonia solution, the slides were stained with eosin solution, dehydrated, and sealed. Histopathological changes were viewed under a light microscope.

### Statistical Analysis

Data are expressed as the mean ± SD and were analyzed by the non-parametric Mann–Whitney *U*-test using SPSS 18.0 (SPSS Inc., USA). Kaplan–Meier survival curves were analyzed using the log-rank (Mantel–Cox) test. Graphs were generated in GraphPad Prism 7.00 (GraphPad Inc., La Jolla, CA, USA). All experiments were performed three times with three technical replicates. Differences between the Nc-1 strain and ΔNcGRA2 strain were considered statistically significant at ^*^p < 0.05 or ^**^p < 0.01, and no significance was denoted as n.s.

## Results

### Characterization of an *N. caninum* Dense Granule Protein NcGRA2

A bioinformatics prediction of NcGRA2 (NCLIV_045650) showed that the signal peptides were located at amino acids 1–23, and no potential transmembrane regions existed (Supplementary Figure 2). Specific primers were designed to amplify the NcGRA2 gene from cDNA using PCR, and a target band of 600 bp was obtained. Then, the NcGRA2 gene was cloned into the pGEX-4T-1 vector, and the positive recombinant plasmid pGEX-NcGRA2 was transformed into Rosetta (DE3). After induction of expression, a 35-kDa protein was obtained. For further characterization of NcGRA2, a rabbit polyclonal antibody against NcGRA2 was also generated.

**Figure 1:**
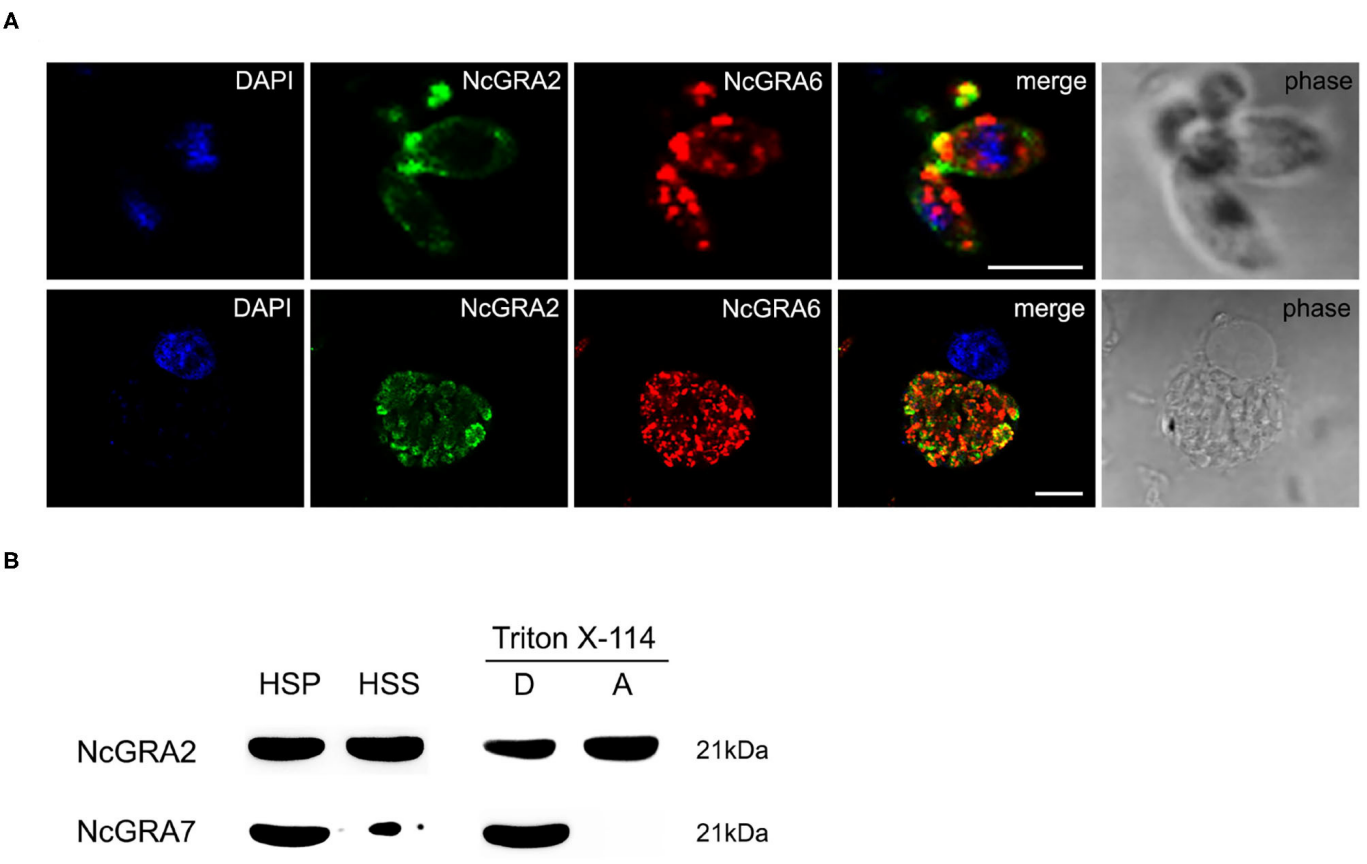
Subcellular localization and morphology analysis of the NcGRA2 protein in parasites and infected cells. **(A)** Immunofluorescence colocalization of NcGRA2 and NcGRA6 proteins in parasites and infected cells. The nucleus was stained with DAPI. The NcGRA2 protein and NcGRA6 protein were labeled with FITC and Cy3, respectively. Bar = 5 μm. **(B)** Morphology analysis of the NcGRA2 protein in parasite-infected cells. HSS represents the soluble cell fraction after high-speed centrifugation. HSP represents the insoluble cell fraction after high-speed centrifugation. The HSPs were then sonicated and centrifuged at high speed, and pellets (P) and supernatants (S) were obtained. The obtained pellets were further dissolved in denaturing agents, and the detergent phase (D) and aqueous phase (A) were obtained. The NcGRA7 protein was used as a control to detect the purity of fractions.

For exploration of the localization of the NcGRA2 protein, immunofluorescence assays were carried out both in the parasites and in infected MDBK cells. As shown in Figure 1A, NcGRA2 could colocalize with NcGRA6 at the posterior end of tachyzoites. For the infected cells, similar to the location of NcGRA6, NcGRA2 was mainly distributed in the IVN structure of PV (38). To further analyze the existing forms of the NcGRA2 protein in the PV, cell fractionation analysis was performed. The purity of fractions was verified using NcGRA7. As shown in Figure 1B, NcGRA2 existed in both the HSS and HSP; moreover, the amounts in the HSS and HSP were approximately the same. This indicated that secreted GRA2 existed in soluble and membrane-associated forms. To further explore the relationship between NcGRA2 and membranes, HSP was then dissolved in Triton X-114 denaturant. After ultracentrifugation, the results showed that HSP could exist in the detergent phase and aqueous phase. These data indicated that NcGRA2 behaved as a transmembrane protein and a membrane-coupled protein.

### Successful Construction of the ΔNcGRA2 Strain

We designed a CRISPR/Cas9 system with site-directed mutagenesis technology for the *N. caninum* NcGRA2 gene, designated pNc-SAG1::CAS9-U6::sgGRA2 (Supplementary Figure 3), based on the *N. caninum* gene editing vector of pNc-SAG1::Cas9-U6::sgUPRT. The full-length DHFR-TS cassette with homologous sequences of the NcGRA2 protospacer adjacent motif was obtained by PCR assay with templates of the *N. caninum* selectable marker pNc-DHFR plasmid. To construct the ΔNcGRA2 strain, the pNc-SAG1::CAS9-U6::sgGRA2 and the DHFR-TS cassette were coelectroporated into Nc-1 tachyzoites, and the ΔNcGRA2 strain was isolated from single clone culture strains after screening under pyrimethamine pressure.

**Figure 2:**
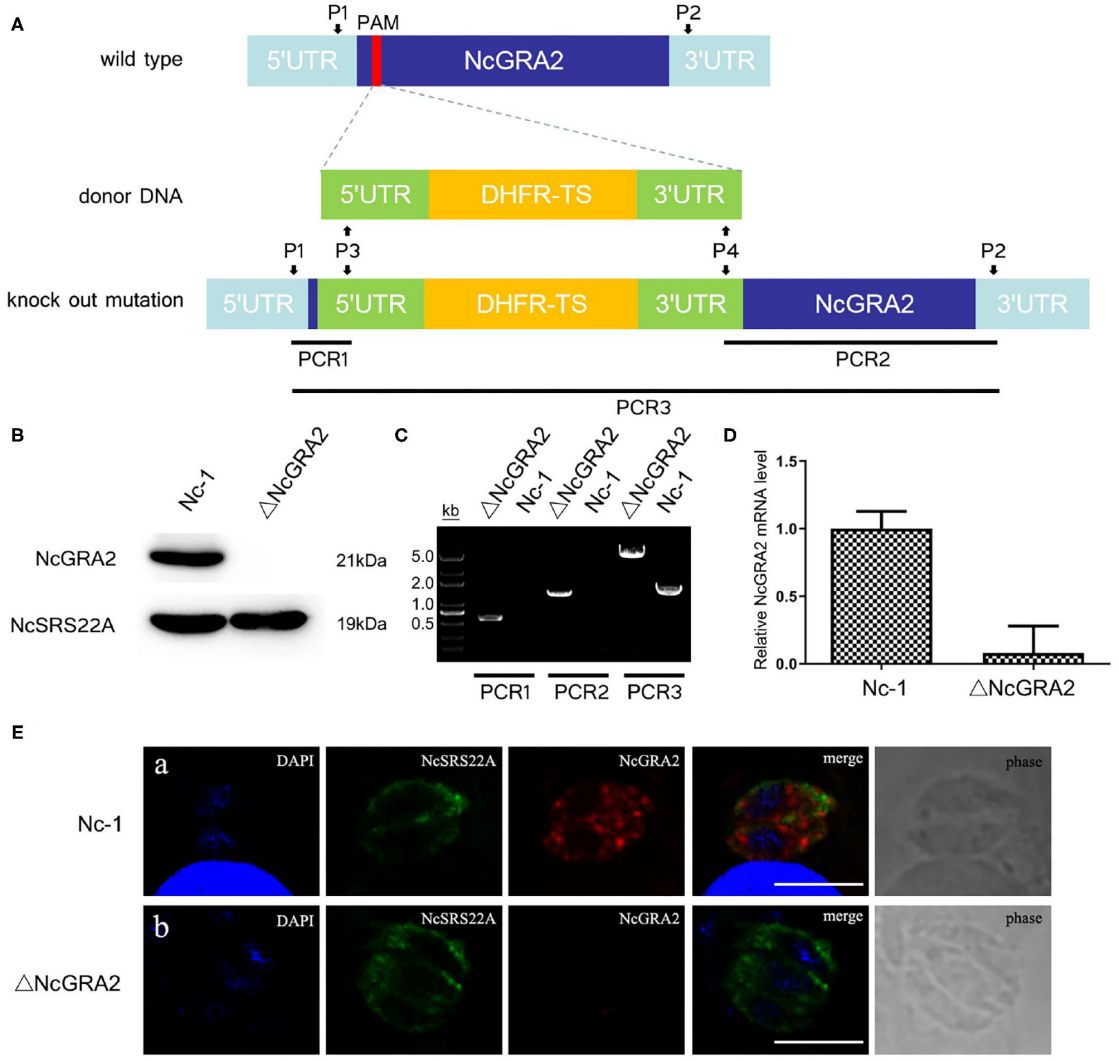
Construction of the ΔNcGRA2 strain. **(A)** Schematic diagram of NcGRA2 gene disruption strain construction and primer design strategies for PCR assessment of the ΔNcGRA2 strain. **(B)** Western blotting assessment of NcGRA2 gene disruption by measuring the protein expression levels of NcGRA2 in the Nc-1 strain and ΔNcGRA2 strain. NcSRS22A was used as control. **(C)** PCR assessment of NcGRA2 gene disruption by measuring the genome levels in the Nc-1 strain and ΔNcGRA2 strain. **(D)** qPCR assessment of NcGRA2 gene disruption by measuring the transcription levels in the Nc-1 strain and ΔNcGRA2 strain. **(E)** Immunofluorescence assessment of NcGRA2 gene disruption by localization analysis of the NcGRA2 protein in the Nc-1 strain and ΔNcGRA2 strain. NcSRS22A was used as control. Bar = 5 μm.

For further assessment of the ΔNcGRA2 strain, the NcGRA2 protein expression level, genome level, and transcriptional level, coupled with the localization of the NcGRA2 protein, were measured. As shown in Figures 2A,B, NcGRA2 protein expression was evaluated in the ΔNcGRA2 strain and Nc-1 strain, and the surface antigen protein NcSRS22A was used as a control. Western blotting analysis indicated that NcSRS22A existed in both strains; however, the NcGRA2 protein existed only in the Nc-1 strain and was absent in the ΔNcGRA2 strain. These results verified that the NcGRA2 protein was disrupted in the ΔNcGRA2 strain.

Next, we determined the NcGRA2 gene at the genome level by PCR amplification. Three PCR products, named PCR1, PCR2, and PCR3, were amplified with primers P1/P3, P2/P4, and P1/P2. As expected, products of PCR1 and PCR2 were both generated only in the ΔNcGRA2 strain, which successfully inserted the DHFR-TS cassette; in contrast, products of 500 and 1,400 bp were absent in the Nc-1 strain. The product size of PCR3 in the NcGRA2 disruption strain was 4,000 bp larger than that in the Nc-1 strain (Figure 2C). After purification of PCR amplification products and sequencing, the results further verified that full-length DHFR-TS cassette had been successfully inserted into the Nc-1 strain accompanied by the NcGRA2 gene disruption.

NcGRA2 gene disruption was also evaluated at the transcriptional level. cDNA from the Nc-1 strain and NcGRA2 disruption strain was prepared, and qPCR assays were carried out. Ct values of the NcGRA2 gene and the housekeeping gene actin were measured, and the mRNA fold change was calculated by 2^−ΔΔCt^. As shown in Figure 2D, the NcGRA2 gene expression level in the ΔNcGRA2 strain group was downregulated in comparison with the control group of the Nc-1 strain. The residual relative mRNA levels of NcGRA2 calculated by 2^−ΔΔCt^ were probably due to the background signals when the target gene was in low concentrations. The results of transcriptional level verified that the NcGRA2 gene was successfully disrupted.

For localization analysis of the NcGRA2 protein, immunofluorescence assays were performed. Infected MDBK cells were coincubated with specific antibodies against NcSRS22A and NcGRA2 and then labeled with different detection antibodies. As shown in Figure 2E, the blue signals represented DAPI-stained nuclei, the green signals represented FITC-conjugated goat anti-mouse IgG (H+L)–stained NcSRS22A protein, and the red signals indicated Cy3-conjugated goat anti-rabbit IgG (H+L)–stained NcGRA2 protein. Both green and red signals existed in the Nc-1 strain; however, the red signal was absent in the ΔNcGRA2 strain. These data indicated that the NcGRA2 protein was not secreted into the PV and that the ΔNcGRA2 strain was successfully constructed.

### Roles of NcGRA2 in *N. caninum* Invasion and Proliferation

**Figure 3:**
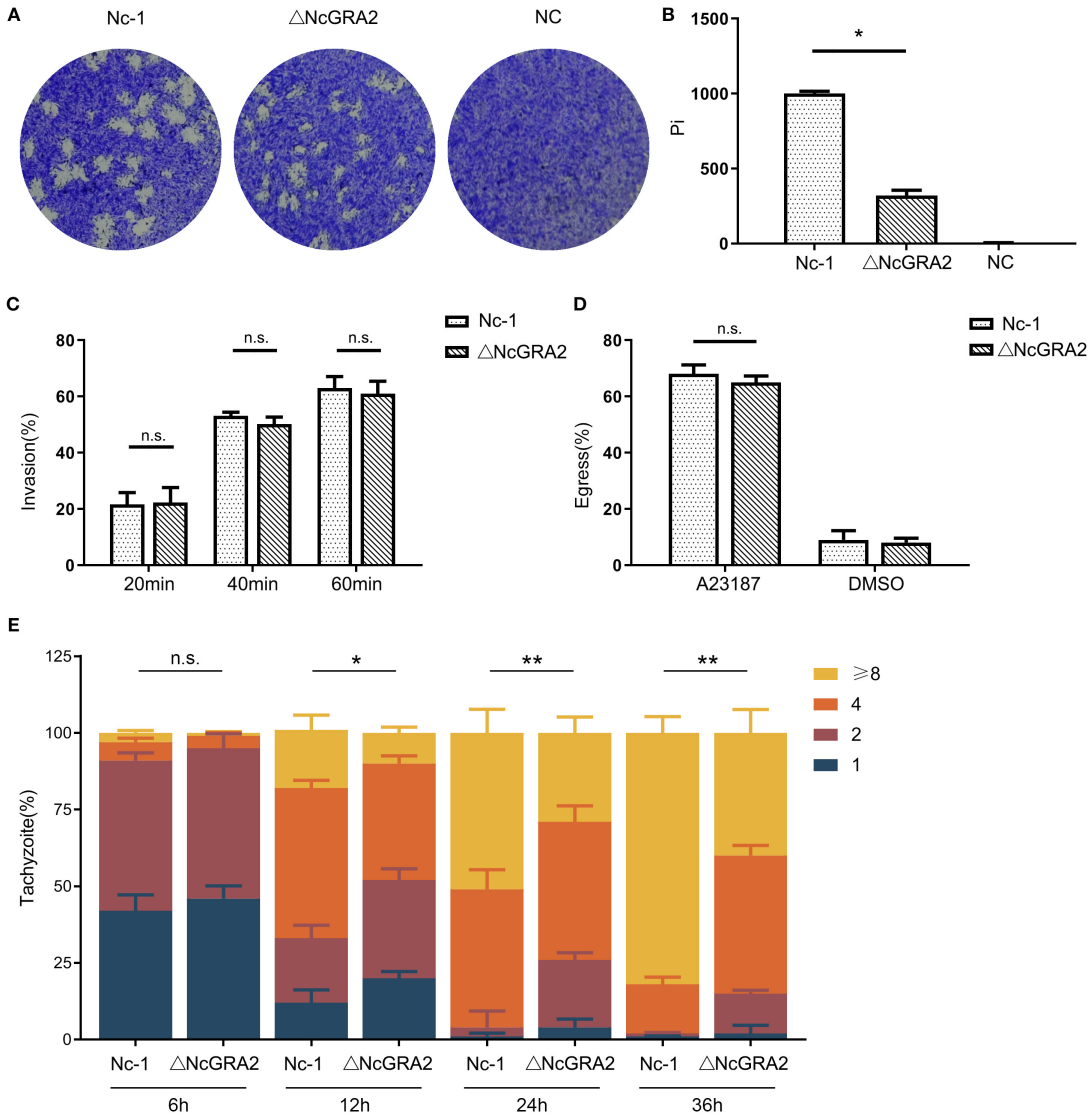
Disruption of the NcGRA2 gene adversely affects virulence *in vitro*. **(A,B)** Plaque assays were carried out by inoculation of parasites onto HFF cells previously coated on six-well plates (80–120 tachyzoites per well), followed by culturing for 7 days. The Nc-1 group was used as a positive control, and the NC group was used as a negative control. After staining with crystal violet, the cells were imaged, and then pixels of plaques (Pi) were measured by randomly selecting no fewer than 20 plaques and counting with pixel points in Photoshop CS5 software. **(C)** Invasion assays were conducted through inoculation of the Nc-1 strain and ΔNcGRA2 strain with an infection dose of 1 × 10^5^ tachyzoites/well onto HFF cells coated on coverslips in 6-well plates and then culturing for the indicated time. Immunofluorescence assays with anti-NcSRS22A and anti-NcGRA2 antibodies were carried out to label the total parasites and extracellular parasites, and then the invasion percentage was calculated. **(D)** Egress assays were performed by inoculation of the Nc-1 strain and ΔNcGRA2 strain onto HFF cells coated on coverslips in six-well plates (1 × 10^5^ tachyzoites/well) and culturing for 48 h. After stimulation with the Ca^2+^ ionophore A23187, immunofluorescence assays with anti-NcGRA2 antibodies were conducted, and the egress percentage was measured from 100 randomly selected vacuoles. The dilution buffer, DMSO, was used as a negative control. **(E)** Intracellular parasite replication assays were measured by pretreatment of HFF cells with the Nc-1 strain and ΔNcGRA2 strain (1 × 10^6^ tachyzoites/well) for 4 h and then culturing for the indicated time. Immunofluorescence assays with anti-NcSRS22A antibodies were performed, and parasite replication was determined by 100 randomly selected vacuoles. *p < 0.05, **p < 0.01, and n.s. represents no significance vs. the Nc-1 group.

After invasion, *N. caninum* enters into host cells, replicates in the PV, egresses out of the PV, and invades uninfected cells. Plaque assays are indicators that comprehensively evaluate the viability of parasites. In this study, to compare the influence of the NcGRA2 gene on the viability of *N. caninum*, host cells were individually inoculated with Nc-1 strain tachyzoites and ΔNcGRA2 strain tachyzoites to carry out plaque assays, and untreated cells were used as a negative control (NC); the pixels of each plaque (pi) were determined. As shown in Figures 3A,B, there were no plaques found in the NC group; in contrast, larger plaques existed in the Nc-1 group, and smaller plaques existed in the NcGRA2 disruption group. Compared with the Nc-1 group, the pi value was obviously downregulated in the ΔNcGRA2 group (^*^p < 0.05).

The difference in plaque formation may be related to invasion, egress, or replication. To determine which critical process significantly influences the viability of parasites in host cells, we first measured the invasion ability after treatment time points of 20, 40, and 60 min. As shown in Figure 3C, along with the prolonged incubation time, the invasion percentages were increased. However, no significance was observed between the Nc-1 strain group and the ΔNcGRA2 strain group, indicating that the NcGRA2 gene was not involved in *N. caninum* invasion ability.

Next, we carried out egress assays to evaluate the influence of the NcGRA2 gene on the viability of parasites in host cells. Monolayer HFF cells were incubated with parasites and then stimulated with a Ca^2+^ ionophore. The egress percentages were calculated by counting the labeled parasites. As displayed in Figure 3D, although the egress percentages in the ΔNcGRA2 strain were lower than those in the Nc-1 strain group, the difference was not significant (*p* > 0.05). In addition, DMSO, the solvent used for A23187, had a relatively low influence on egress ability. Thus, we inferred that the NcGRA2 gene also had no function in *N. caninum* egress out of PV.

The proliferation assays were carried out by counting the amounts of parasites in each PV after different inoculation time points of 6, 12, 24, and 36 h. As shown in Figure 3E, at the initial infection stage, a large number of vacuoles contained one or two parasites; moreover, no significant difference was found in these two groups (*p* > 0.05). At 12 h, the numbers of parasites became larger in both groups, with more than half of the vacuoles containing two or four parasites; in addition, the total number of parasites in the Nc-1 group was obviously larger than that in the ΔNcGRA2 group (^*^p < 0.05). As the infection time increased, the proliferation rate increased in the Nc-1 group, accompanied by most vacuoles containing eight parasites; in contrast, only four parasites were present in most vacuoles of the ΔNcGRA2 group. The total number of parasites in the ΔNcGRA2 group was significantly less than that in the Nc-1 group at both 24- and 36-h time points (^**^p < 0.01). Overall, these data illustrated that the NcGRA2 gene could control *N. caninum* replication in the PV and influence the viability of parasites in host cells.

### Disruption of the NcGRA2 Gene Decreased *N. caninum* Virulence

**Figure 4:**
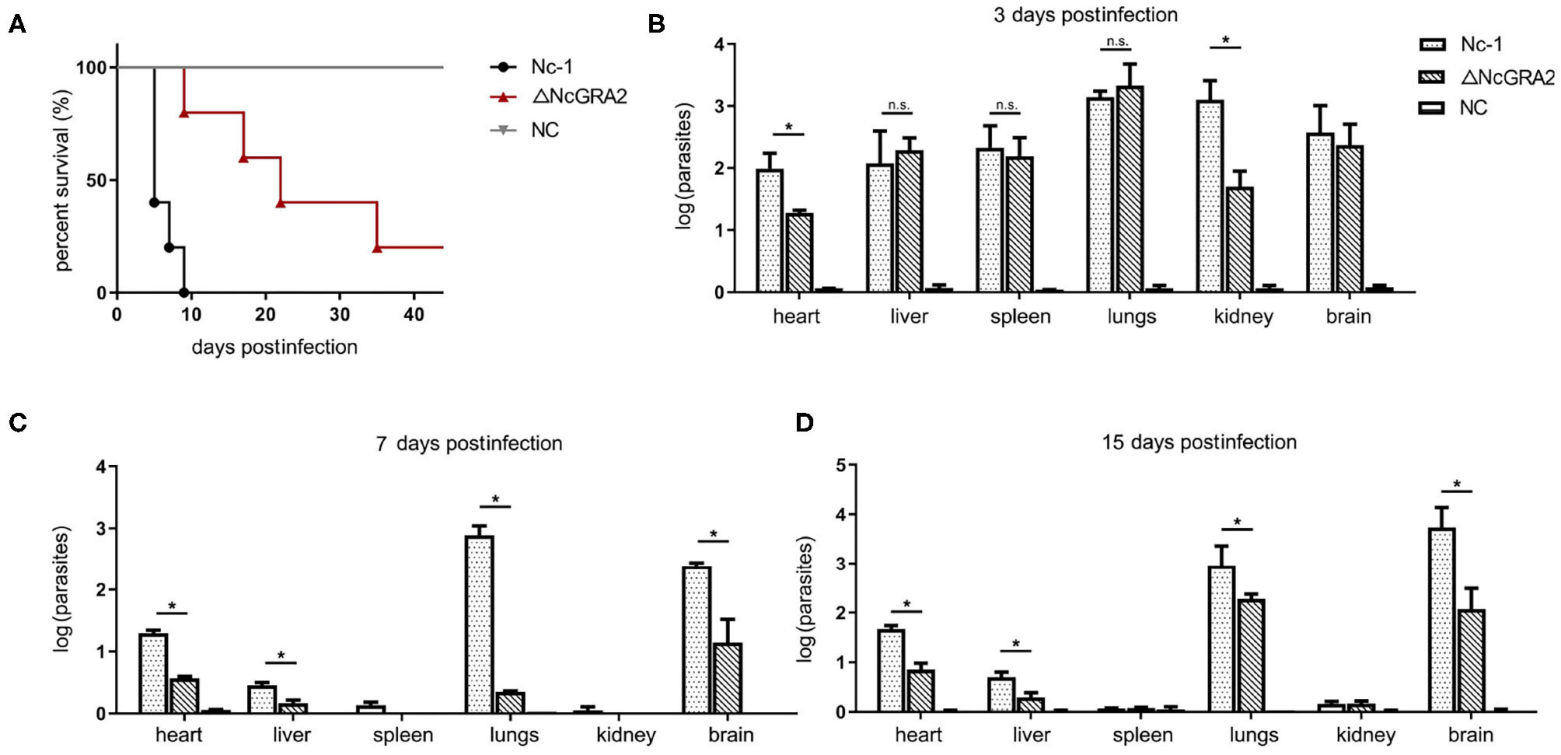
Disruption of the NcGRA2 gene decreases the virulence *in vivo*. **(A)** Monitoring of mouse survival after injection with the Nc-1 strain and ΔNcGRA2 strain. The female BALB/C mice were injected i.p. with 2 × 10^7^ tachyzoites per mouse. The survival time were monitored for 40 days. The NC group was treated with an equal volume of PBS. **(B**–**D)** Determination of parasite burden in different tissues at the indicated time. The female BALB/C mice were injected i.p. with 2 × 10^6^ tachyzoites per mouse. Then, the mice were humanely euthanized, and heart, liver, spleen, lung, kidney, and brain tissues were isolated. The NC group was treated with equal volume of PBS. Parasites burden was determined by qPCR. *p < 0.05, and n.s. represents no significance.

Virulence *in vivo* was comprehensively evaluated by establishing infection models. For the acute infection, mice were injected i.p. with a high dose of the Nc-1 strain or NcGRA2 disruption strain (2 × 10^7^ per mouse) and monitored for the survival days. As shown in Figure 4A, inoculation of Nc-1 strain tachyzoites caused mice to begin to die at day 6, and all the mice died by day 9; however, inoculation of ΔNcGRA2 strain tachyzoites caused 20% of mice to die at day 9, and 20% of mice were still alive at day 40. The survival percentage in the ΔNcGRA2 group was obviously higher than that in the Nc-1 group (*p* = 0.0041). These data indicated that the NcGRA2 gene played roles in the pathogenicity of *N. caninum* and that knockout of the NcGRA2 gene could prolong the survival time of the hosts.

To compare the influence of the Nc-1 strain and the NcGRA2 disruption strain tachyzoites on tissue damage, non-lethal doses of tachyzoites (2 × 10^6^ tachyzoites per mouse) were inoculated i.p. into mice. Tissues were separated, and the amounts of parasites were determined at 3, 7, and 15 dpi. As shown in Figure 4B, at the initial infection stage of 3 dpi, all the separated tissues, including hearts, livers, spleens, lungs, kidneys, and brains, had relatively high parasite burdens in both groups. Moreover, parasite burdens were significantly decreased in the ΔNcGRA2 group compared with the Nc-1 group in hearts and kidneys (^*^p < 0.05); however, there were no significant differences in other tissues (*p* > 0.05). At 7 and 15 dpi, lungs and brains were the major parasite reserve organs for the Nc-1 strain infection group; in contrast, the NcGRA2 disruption group had low levels of parasites, especially in the lungs (^*^p < 0.05). Similar to heart and liver tissues, although at low levels of parasite burden when compared with the initial infection stage, the parasite amounts were significantly lower in the ΔNcGRA2 group than in the Nc-1 group (^*^p < 0.05). Low levels of parasite burdens were detected in the spleen and kidney groups at 7 and 15 dpi (Figures 4C,D). Overall, the lung and brain tissues were the storage organs of *N. caninum*, and disruption of the NcGRA2 gene could obviously decrease the parasite levels in targeted organs and may further lower its pathogenicity.

**Figure 5:**
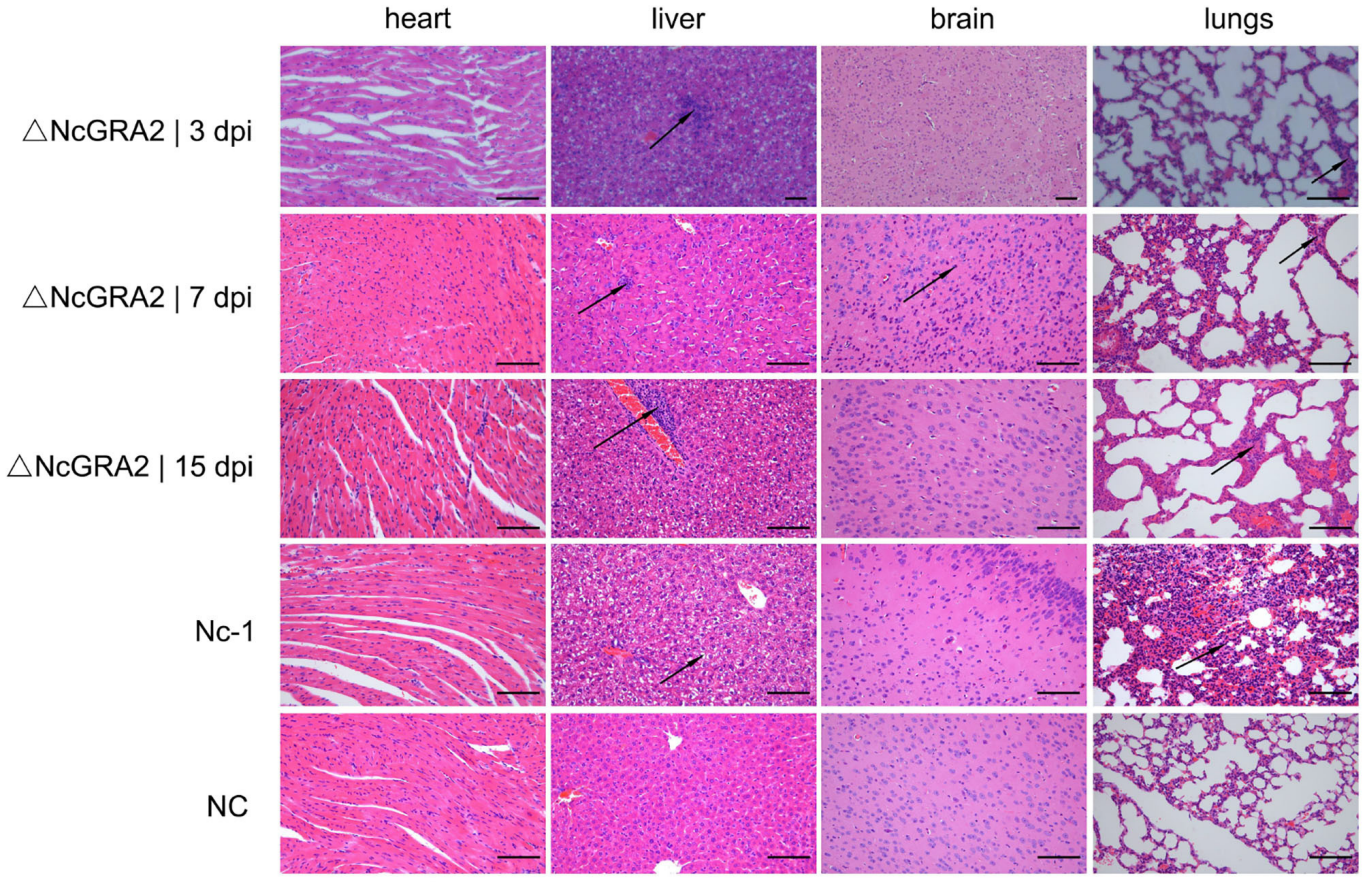
Disruption of the NcGRA2 gene relieves histopathological changes in the targeted tissues of infected mice. The female BALB/C mice were injected i.p. with 2 × 10^6^ tachyzoites per mouse and then euthanized at the indicated time. The targeted heart, liver, brain, and lung tissues were isolated, and HE staining assays were conducted. The Nc-1 strain group was used as a positive control, and the NC group was used as a negative control. dpi represent days postinfection. Bar = 100 μm. Arrows indicate typical histopathological changes.

Histopathological changes were detected in the targeted organs. Compared with the NC group at 15 dpi, Nc-1 strain–infected mice at 15 dpi displayed significantly thickened alveolar mass in lung tissues, obvious inflammatory cell infiltration and small focal necrosis in liver tissues, and glial cell proliferation in brain tissues. For the ΔNcGRA2 group, histopathological changes in lung tissues displayed slightly widened alveolar masses at 3, 7, and 15 dpi. Liver tissues showed slight inflammatory cell infiltration surrounding the central veins throughout the infection stage and partial denaturation at 15 dpi. Brain tissues only displayed glial cell proliferation at 7 dpi. Furthermore, no changes in heart tissues were found in any of the infection groups (Figure 5). These data indicated that the NcGRA2 gene played roles in regulating *N. caninum*–induced tissue damage, especially in target organs of the lungs. Disruption of the NcGRA2 gene could decrease the pathogenicity of *N. caninum*.

## Discussion

During *N. caninum* invasion into host cells, many different kinds of secretory antigens are involved in this process and play various biological functions. In our previous studies, we found that GRA proteins increased most significantly compared with other secretory antigens of MICs and ROPs (unpublished data). This illustrated that GRAs were the most important components of PV and may play critical roles in the proliferation of *N. caninum*. In *T. gondii*, based on the different positions where they are released, GRAs are divided into three forms: soluble proteins, such as GRA1 targeted the lumen of PV; integral membrane proteins, such as GRA3 localized to the delimiting membrane of the PV; and soluble and membrane-associated proteins, such as GRA2 situated in the IVN, which belongs to the reticulum of membranous tubules (39). Although the exact mechanism remains unclear, the amino acid sequence analysis of some GRAs existing in the transmembrane region suggests that they may be key factors associated with membranes. It has been verified that tubulogenic proteins of GRA2 and GRA6 can stabilize the IVN structure in the PV of *T. gondii* and display great significance in parasite proliferation (21, 25, 40, 41). The roles of NcGRA6 have been elucidated (29, 38); however, research on the roles of NcGRA2 in *N. caninum* is limited. In this study, NcGRA2 sequences were obtained from the *N. caninum* Nc-1 strain and shared 100% amino acid sequence homology with previously published NcGRA2 in the *N. caninum* NC-Liverpool strain (NCLIV_045650). In Ellis' published article, the NcGRA2 amino acid sequences were released into GenBank (AAG28489.1), and there are 100% homology between NcGRA2 amino acid sequences in NCLIV_045650 and AAG28489.1 (26). A bioinformatics analysis showed that only signal peptides existed and that there were no transmembrane regions in the NcGRA2. In contrast, HSP and HSS analysis showed that NcGRA2 existed in the PV in soluble and membrane-bound forms. An in-depth analysis of HSP by dissolving in Triton X-114 showed that NcGRA2 associated with PVM as a transmembrane protein and a membrane-associated protein. Interestingly, our prediction analysis showed that there was no transmembrane region. In *T. gondii*, GRA2 was embedded in the IVN of PV through the structures of two AAHs (19, 21). Thus, the protein structure of NcGRA2 was further analyzed in ToxoDB, which displayed that NcGRA2 and TgGRA2 had similar AAH distribution, inferring that the transmembrane structure in *N. caninum* may also be formed through embedding in the IVN of PV with the aid of AAHs. To further characterize the localization of NcGRA2, immunofluorescence assays were carried out by assessing colocalization with NcGRA6. Previous studies showed through immunoelectron microscopy analysis that NcGRA6 was specifically localized in the GRAs of *N. caninum* tachyzoites and IVN of PV (29). Most GRAs could colocalize with NcGRA6 in tachyzoites except for NcGRA1, NcGRA3, NcGRA5, or NcGRA12, which may share different protein secretory pathways or synthesis locations (unpublished data). Moreover, most GRAs colocalized with NcGRA6 in the PV of infected MDBK cells and were involved in the composition of the IVN, except for NcGRA5, which was an integral transmembrane protein localized in the PVM and not involved in the composition of the IVN (unpublished data). Thus, NcGRA6 was used as an indicator to preliminarily explore the localization of NcGRA2 in tachyzoites and infected cells. From the colocalization analysis in the tachyzoites, the NcGRA2 protein was mostly colocalized with NcGRA6, which indicates that NcGRA2 could be secreted from GRAs of *N. caninum*. From the colocation analysis in the infected cells, NcGRA2 protein was completely colocated with NcGRA6, indicating that NcGRA2 was also a constituent of the IVN. Similar to our results, the NcGRA2 location in bradyzoites was within the vacuoles; however, it was also found in the vacuole periphery, although not pronounced, which was different from that in tachyzoites (27). In *T. gondii*, results of GRA2 gene disruption and TgGRA2 gene complement showed that the N-terminal domain and the three main amphoteric α-helices were all necessary for IVN formation, which indicated that TgGRA2 involved in the formation of IVN (25, 42). Our results found that NcGRA2 was also localized in the IVN; however, its roles on the IVN formation still need to be further determined in the future.

To further determine the gene function of NcGRA2 in *N. caninum*, CRISPR/Cas9 technology was used to construct the NcGRA2 disruption strain. For the study of protozoon of the phylum Apicomplexa, the CRISPR/Cas9 system was used to construct a model organism *T. gondii* gene knockout strain in 2014 for the first time. The CRISPR/Cas9 system mainly contains two parts: the Cas9 dsDNA nuclease and guide RNA, a cofactor used to specifically select targeted genes and cause breaks in dsDNA (43). Research has found that there are many repair methods for double-stranded breaks, including HDR and NHEJ, in *T. gondii* (44, 45). The HDR pathway is the most classical method; however, it is a repair mechanism with a lengthy period and a low efficiency and is limited by a relatively long homologous arm. Therefore, a relatively more efficient method of NHEJ has been widely applied in the study of *T. gondii*. Using the CRISPR/Cas9 system in *T. gondii* significantly improved the gene editing efficiency while avoiding the drawbacks of traditional manipulation (46–49). *N. caninum* also belongs to the phylum Apicomplexa and shares relatively high homology with *T. gondii*. Previous protein function research on *N. caninum* mainly used the homologous recombination method (33), which shared a lower gene editing efficiency and higher failure rate. Testing the gene functions in *N. caninum* was previously severely compromised by limited technologies until the *T. gondii* CRISPR/Cas9 system was used to generate the NcGRA7 gene disruption strain for the first time in 2018 (50). Considering the experience obtained with *T. gondii*, we made initial attempts to construct an NcGRA2 gene disruption strain with the *T. gondii* CRISPR/Cas9 system (51); however, we failed, with a low gene editing efficiency observed. Next, we found that Yang et al. attempted to utilize the strong RNA polymerase III *N. caninum* U6 promoter instead of *T. gondii* to efficiently knock out the NcGRA17 gene (52). Moreover, Zhao et al. used pNc-SAG1::CAS9-U6::sgUPRT containing the *N. caninum* U6 promoter to successfully generate an NcGRA6 gene knockout strain (29). In this study, based on the *N. caninum* U6 promoter and sgRNA sequences for NcGRA2, we constructed a pNc-SAG1::CAS9-U6::sgGRA2 and then generated a ΔNcGRA2 strain through coelectrotransforming the gene editing plasmid and DHFR-TS cassette into Nc-1 tachyzoites. sgGRA2 is regulated by the *N. caninum* U6 promoter, and Cas9 transcription is regulated by the SAG1 promoter and terminator of *N. caninum*. The gene editing system disrupts the NcGRA2 gene by insertion of the DHFR-TS marker. Under pyrimethamine pressure screening and single clone culturing, an NcGRA2 gene disruption strain was finally obtained. Overall, the CRISPR/Cas9 system could make gene function research available for a variety of parasitic protozoa with a high efficiency (53–57).

Although limited research has focused on the protein functions of *N. caninum*, studies on the protein functions of *T. gondii* provide a reference. In *T. gondii*, TgGRA2 expression could be detected both in tachyzoites at different growing stages and in the IVN of the PV. Disruption of the TgGRA2 gene reduced IVN formation by inhibiting Rab11A vesicle formation and influenced the pathogenicity of *T. gondii* (19, 41). To date, only two studies have focused on GRA function research in *N. caninum* virulence by constructing *N. caninum* knockout strains, namely, GRA6 and GRA17 (29, 52). Inoculation of the ΔNcGRA6 Nc-1 strain prolonged the mouse survival time to 24 days compared with the wild type Nc-1 strain with a survival time of 8 days (29). The ΔNcGRA17-infected mice were all alive during the monitored 60 days; in contrast, all the mice died in the wild type Nc-1 strain group (52). However, the function of NcGRA2 has not been fully clarified. The association of NcGRA2 and the pathogenicity of *N. caninum* need to be determined. In this study, we showed that disruption of the NcGRA2 gene reduced tachyzoite proliferation and decreased pathogenicity in mice, accompanied by higher survival rates and lower tissue damage and parasite burden. For the evaluation of egress ability, similar to NcGRA6, the ΔNcGRA2 group shared no significant difference compared with that in the wild-type group (29). Results illustrate that these two NcGRA proteins do not involve in the egress process in tachyzoite-infected cells. Overall, these data highlighted the roles of NcGRA2 in the virulence of *N. caninum*.

## Conclusions

We characterized the *N. caninum* GRA protein NcGRA2, constructed an NcGRA2 gene disruption strain based on the Nc-1 strain with the CRISPR/Cas9-directed genome editing system, and highlighted the roles of NcGRA2 in *N. caninum* virulence through *in vivo* and *in vitro* assays.

## Data Availability Statement

The original contributions presented in the study are included in the article/Supplementary Material, further inquiries can be directed to the corresponding author/s.

## Ethics Statement

The animal study was reviewed and approved by Animal Welfare and Research Ethics Committee of Jilin University.

## Author Contributions

JD designed the research. JD and NZ conducted the research. PG, JL, LC, XW, XL, and JY analyzed the data. JD and PZ wrote the manuscript. PG and XZ directed the project. All authors have read and approved the manuscript.

## Conflict of Interest

The authors declare that the research was conducted in the absence of any commercial or financial relationships that could be construed as a potential conflict of interest.
