# Supplementary material for: Disruption of Dense Granular Protein 2 (GRA2) Decreases the Virulence of Neospora caninum
Source: Front Vet Sci. 2021 Feb 19;8:634612. doi: 10.3389/fvets.2021.634612 (PMC7933011; doi:10.3389/fvets.2021.634612)

FIGURE S1 ***N. caninum*** **gene editing plasmid map of pNc-SAG1::CAS9-U6::sgUPRT.**

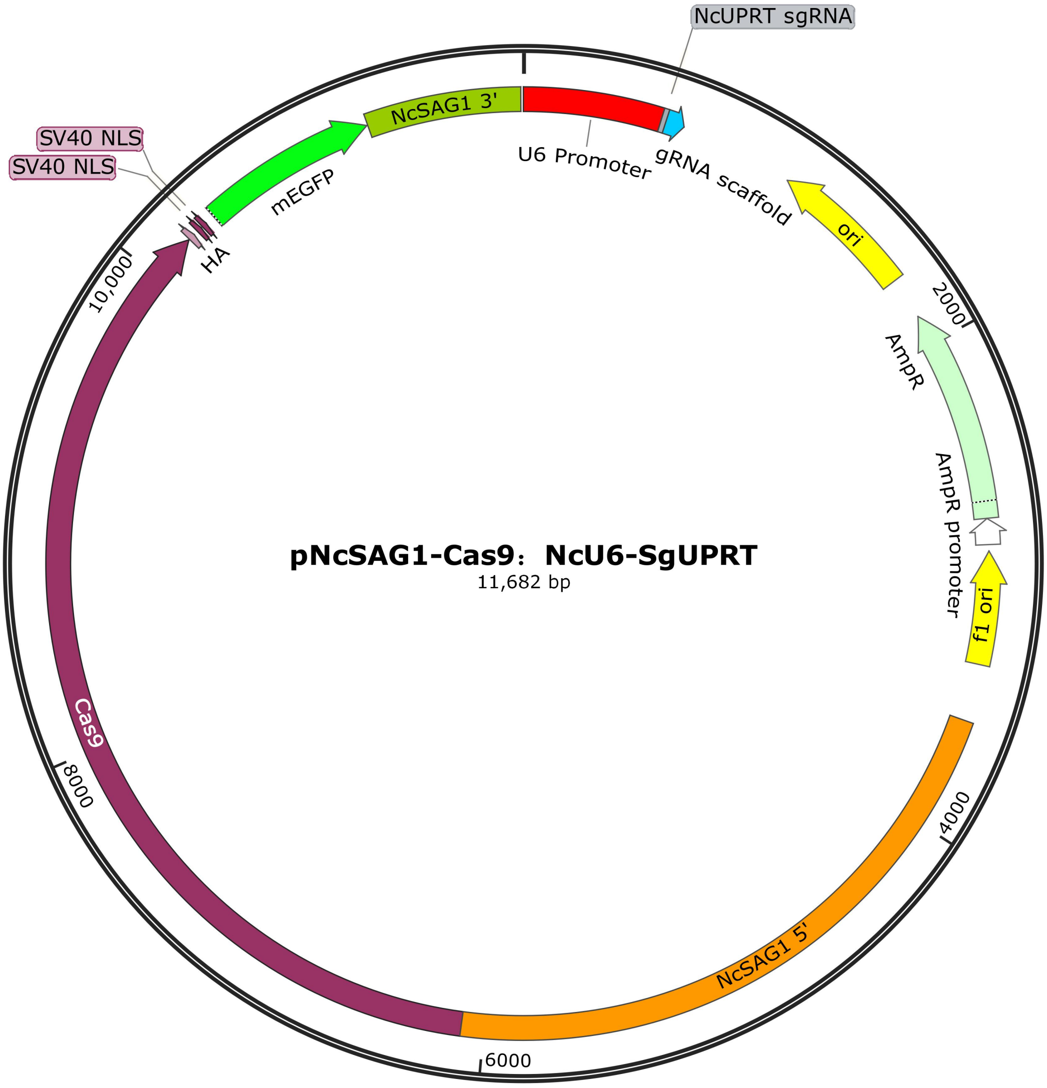

FIGURE S2 Bioinformatics prediction of NcGRA2.

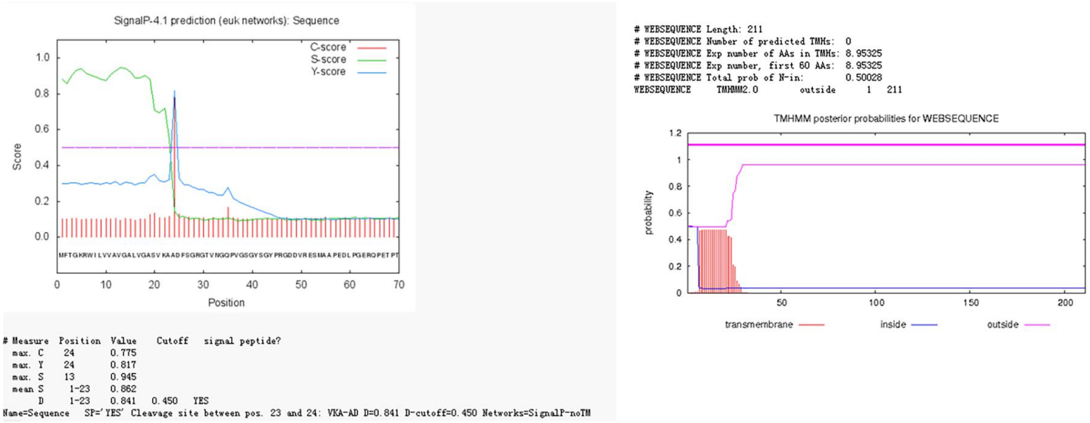

FIGURE S3 ***N. caninum*** **gene editing plasmid map of pNc-SAG1::CAS9-U6::sgGRA2.**

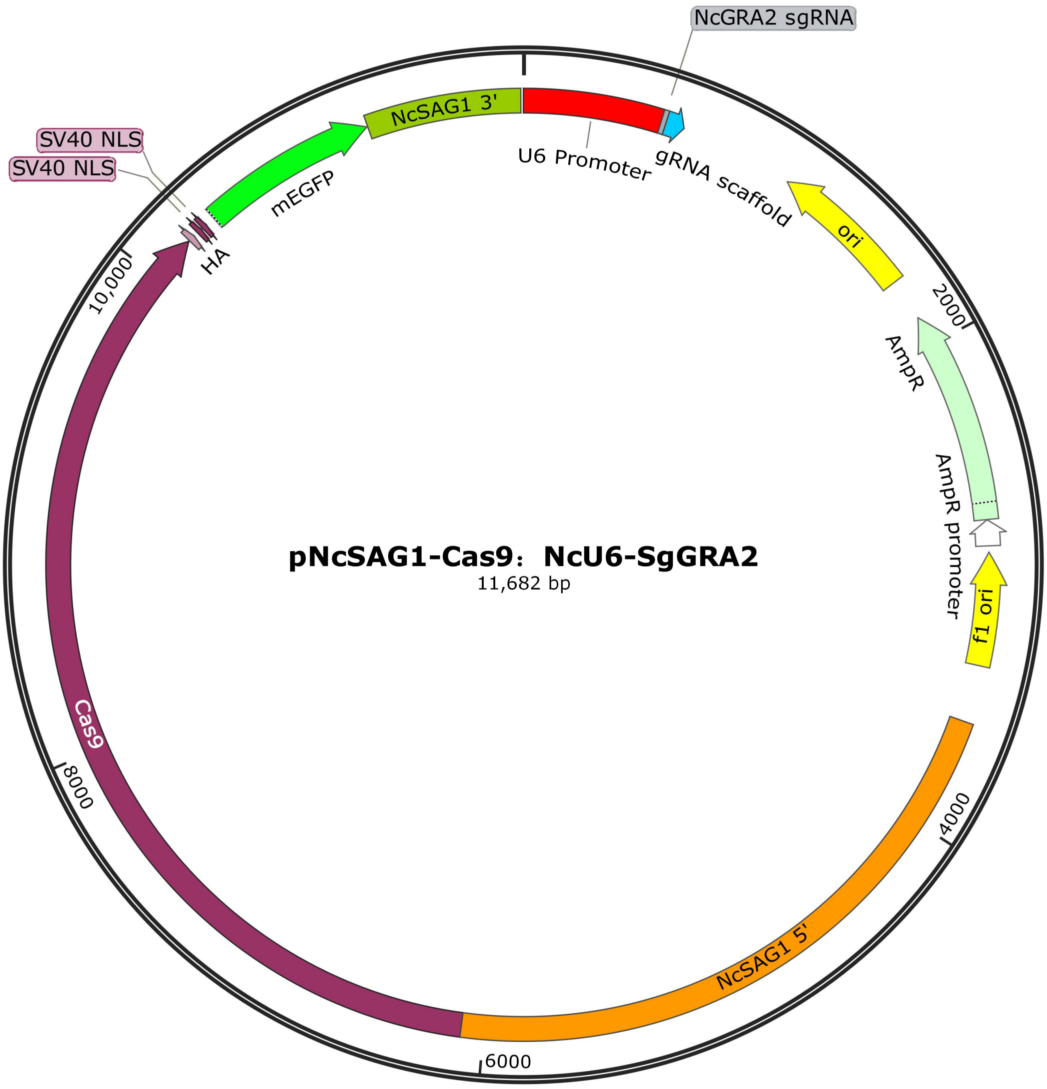

Supplement: Supplementary file 1 [file Presentation_1.pdf]
