# Supplementary material for: Disruption of Dense Granular Protein 2 (GRA2) Decreases the Virulence of Neospora caninum
Source: Front Vet Sci. 2021 Feb 19;8:634612. doi: 10.3389/fvets.2021.634612 (PMC7933011; doi:10.3389/fvets.2021.634612)
Supplement: Supplementary file 2 [file Presentation_2.pdf]

Image 1 Original western blot image of figure 2 B-NcGRA2.

Image 2 Original western blot image of figure 2 B-NcSRS22A.

Image 3 Original western blot image of figure 1 B-NcGRA2-HSP-HSS.

Image 4 Original western blot image of figure 1 B-NcGRA2-Triton X-114.

Image 5 Original western blot image of figure 1 B-NcGRA7-HSP-HSS.

Image 6 Original western blot image of figure 1 B-NcGRA7-Triton X-114.

-
